# Machine Learning Framework for Ovarian Cancer Diagnostics Using Plasma Lipidomics and Metabolomics

**DOI:** 10.3390/ijms26146630

**Published:** 2025-07-10

**Authors:** Alisa Tokareva, Mariia Iurova, Natalia Starodubtseva, Vitaliy Chagovets, Anastasia Novoselova, Evgenii Kukaev, Vladimir Frankevich, Gennady Sukhikh

**Affiliations:** 1 V.I. Kulakov National Medical Research Center for Obstetrics, Gynecology and Perinatology, Ministry of Healthcare of Russian Federation, 117997 Moscow, Russia; a_tokareva@oparina4.ru (A.T.); n_starodubtseva@oparina4.ru (N.S.); v_chagovets@oparina4.ru (V.C.); a_novoselova@oparina4.ru (A.N.); e_kukaev@oparina4.ru (E.K.); v_frankevich@oparina4.ru (V.F.); g_sukhikh@oparina4.ru (G.S.); 2 V.L. Talrose Institute for Energy Problems of Chemical Physics, N.N. Semenov Federal Research Center for Chemical Physics, Russian Academy of Sciences, 119334 Moscow, Russia; 3 Moscow Center for Advanced Studies, 123592 Moscow, Russia; 4 Laboratory of Translational Medicine, Siberian State Medical University, 634050 Tomsk, Russia; 5 Department of Obstetrics, Gynecology, Perinatology and Reproductology, Institute of Professional Education, Federal State Autonomous Educational Institution of Higher Education, I.M. Sechenov First Moscow State Medical University of the Ministry of Health of the Russian Federation, 119991 Moscow, Russia

**Keywords:** ovarian tumor, plasma, metabolome, machine learning, feature selection, MLP, XGBoost, CNN, neural network

## Abstract

Ovarian cancer (OC), the third most common gynecologic malignancy, exhibits distinct metabolic alterations that could enable early detection via liquid biopsy. We developed an advanced machine learning pipeline integrating lipidomics (HPLC-MS, positive/negative ion modes) and NMR-based metabolomics to analyze plasma samples from 229 subjects, including 103 serous OC patients, 107 benign cases, and 19 healthy controls. By systematically evaluating feature selection methods and machine learning architectures, we identified optimal biomarker combinations for OC detection. Convolutional Neural Network (CNN) model based on Mann–Whitney-selected features demonstrated strong discriminatory power (81% accuracy) in distinguishing malignant from benign cases, while Extreme Gradient Boosting (XGBoost) combined with Support Vector Machine-Recursive Feature Elimination (SVM-RFE) achieved exceptional performance (96% accuracy) in differentiating benign from control samples. For multiclass classification, XGBoost with Kruskal–Wallis-selected features achieved 77% accuracy, while one-versus-one CNN models utilizing Mann–Whitney-selected features attained 78% accuracy, demonstrating optimal performance among tested approaches. The complementary strengths of deep learning and ensemble methods underscore their potential for tailored diagnostic applications. While clinical implementation requires further standardization, these findings provide both a methodological framework for metabolic biomarker discovery and biological insights into OC pathophysiology, paving the way for integrated multi-omics approaches in gynecologic oncology.

## 1. Introduction

Ovarian cancer (OC) remains a significant global health challenge, with GLOBOCAN reporting 69,472 new cases and 46,232 deaths across Europe in 2022 alone [1]. This malignancy is particularly insidious because it is frequently diagnosed at advanced stages, with approximately 70% of cases detected after regional or distant metastasis has occurred. Current diagnostic protocols typically identify ovarian tumors during routine gynecological examinations, where accurate malignancy determination becomes crucial for clinical decision-making and treatment stratification. The standard diagnostic approach includes the Risk of Malignancy Index (RMI) and Risk of Ovarian Malignancy Algorithm (ROMA), which combine ultrasound findings, menopausal status, and serum CA125 levels [2]. Although CA125 has been the gold standard biomarker for decades, its limited sensitivity (50–60% for early-stage disease) and specificity (compromised by benign conditions) have spurred research into alternative molecular signatures [3].

Recent advances in molecular diagnostics have identified promising alternatives to CA125, including protein panels (particularly CA15-3, CA 19-9, HE4, and hCG [4,5,6,7,8]), circulating miRNAs (such as miR-200 family members [8,9]), and small molecules (notably lysophospholipids and acylcarnitines [10,11]). The emerging field of metabolomics has shown great promise, with studies demonstrating that tumor-specific metabolic reprogramming [12,13] produces distinct signatures in both tissue biopsies and biological fluids [14,15,16,17]. These -omics approaches offer potential for earlier detection and more accurate differentiation between benign and malignant states, with some recent studies reporting area under the curve (AUC) values exceeding 0.90 in validation cohorts [18,19].

However, -omics research (encompassing metabolomics, proteomics, and lipidomics) faces the persistent challenge of the “curse of dimensionality” [20,21]. This phenomenon, where datasets contain orders of magnitude more features (p) than samples (n), poses significant statistical challenges for robust biomarker discovery [22,23,24,25]. Modern analytical pipelines address this through sophisticated computational strategies, including three primary feature selection approaches: filter methods (employing univariate statistical thresholds), wrapper methods (using iterative classifier performance), and embedded methods (with built-in feature selection like LASSO (least absolute shrinkage and selection operator) regression) [21,26,27]. Recent methodological innovations have demonstrated that hybrid approaches—particularly ensemble feature selection combining multiple methods [28,29,30,31,32] or consensus analysis across different platforms [14]—can improve biomarker reliability. Similarly, integrating multiple classification algorithms (including Random Forest (RF), Support Vector Machines (SVM) with non-linear kernels, and regularized regression models) has shown promise in overcoming individual method limitations [15,33,34]. Deep learning approaches are also gaining traction, with convolutional neural networks (CNN) achieving notable success in image-based OC diagnostics [26,35].

This study aims to develop and validate robust classification models capable of distinguishing between healthy controls, benign ovarian tumors, and OC. Our approach will incorporate lipidomic (liquid chromatography–mass spectrometry, HPLC-MS) and metabolomic (nuclear magnetic resonance, NMR) data for more than 200 patients while implementing advanced feature selection and machine learning techniques to address the high-dimensionality challenge inherent in -omics datasets.

## 2. Results

### 2.1. Clinical Characteristics of Study Participants

Plasma lipid and metabolite profiles were comprehensively analyzed across three well-characterized cohorts: patients with serous ovarian carcinoma (*n* = 103), benign ovarian tumors (*n* = 107), and healthy controls (*n* = 19) to identify potential diagnostic biomarkers. As detailed in Table 1, the OC cohort exhibited distinct histopathological characteristics, with high-grade serous carcinoma representing the predominant subtype (57%, *n* = 59). The remaining cases comprised borderline tumors (27%, *n* = 28) and low-grade carcinomas (16%, *n* = 16). Among high-grade cases classified by International Federation of Gynecology and Obstetrics (FIGO) staging, the distribution was as follows: stage I (*n* = 5, 8.5%), stage II (*n* = 6, 10.2%), stage III (*n* = 44, 74.6%), and stage IV (*n* = 4, 6.8%).

### 2.2. Plasma Lipidome/Metabolome Data

Our HPLC-MS analysis identified 280 distinct lipid species, representing diverse biochemical classes. Lipid profile of plasma included 49 ether-linked glycerophospholipids (PC O-/P- and PE O-/P-), 45 diacylphosphatidylcholines (PC), and 36 oxidized lipids (OxL), along with 27 sphingomyelins (SM), 13 lysophospholipids (LPC, LPE), 15 monogalactosyldiacylglycerols (MGDG), 9 ceramides (Cer), 7 cholesterol esters (CE), and 54 triglycerides (TG). Detection specificity varied by lipid class: TG and CE were only observable in positive ion mode, while oxidized glycerophospholipids and diacylphosphatidylinositols required negative ion mode analysis. Cer and LPE demonstrated optimal detection in negative mode, in contrast to LPC, PC O-/P-, and SM, which were reliably detected in both ionization modes.

The NMR analysis expanded our metabolic characterization, identifying 33 crucial metabolites spanning several biochemical categories. These included amino acids such as alanine, arginine, and glutamine, alcohols like ethanol and myoinositol, various ketoacids including 2-hydroxybutyrate, and carboxylic acids such as citrate and lactate. Beyond individual metabolites, we derived 36 clinically significant metabolite ratios that provide insights into critical metabolic pathway activities.

The blood metabolic profile changes significantly with age: a total of 106 metabolites showed statistically significant associations with age (Supplementary Table S8). Among these, seven lipid species—LPC 18:2, LPC O-16:0, PC O-16:1/18:1, LPC P-16:0, PC 18:2_22:6, PC P-18:0/18:1, and PE P-18:0/18:2—exhibited negative correlations with age (coefficient < −0.20). Conversely, twelve metabolites—PC 16:0_16:0, PC 16:0_18:3, TG 16:0_18:1_18:1, DG 18:1_18:1, MGDG 18:1_22:6, PE 18:1_20:0, SM d18:1/18:0, TG 18:0_18:1_18:2, OxPE 16:0_18:2(OOO), SM d20:0/16:0, PE 16:0_22:6, and PS 16:0_20:3—showed positive correlations with age (coefficient > 0.20).

Through integrated analysis of both HPLC-MS and NMR datasets, we performed rigorous feature selection followed by advanced multivariate statistical approaches. This comprehensive strategy enabled identification of the most biologically and clinically relevant molecular signatures, establishing a robust foundation for subsequent biomarker discovery and pathway analysis [36].

### 2.3. Feature Selection

#### 2.3.1. Comparative Performance and Stability of Feature Selection Methods in Binary Classification

The most stable method for binary comparisons was the SVM-Recursive Feature Elimination (RFE), as shown in Table 2. This method also demonstrated the highest stability due to its large final marker sets: 85 markers for distinguishing benign versus malignant ovarian tumors, 13 for benign tumors versus controls, and 14 for malignant tumors versus controls (Table S1). In contrast, LASSO and Boruta exhibited extremely low stability, failing to produce consistent marker sets (Table S1 and Table 2).

Principal component (PC) space based on Mann–Whitney-selected markers yielded the optimal clustering results, with the lowest values for Hubert–Levin’s C-index and Davies–Bouldin index (Table 2). The SVM-RFE marker space performed worse than both the Mann–Whitney and Welch methods in terms of cluster compactness and separation. However, Orthogonal Projection on Latent Structures-Discriminant Analysis (OPLS-DA) markers achieved the highest Calinski–Harabasz pseudo-F statistic, followed closely by SVM-RFE markers (Table 3).

**Figure 1:**
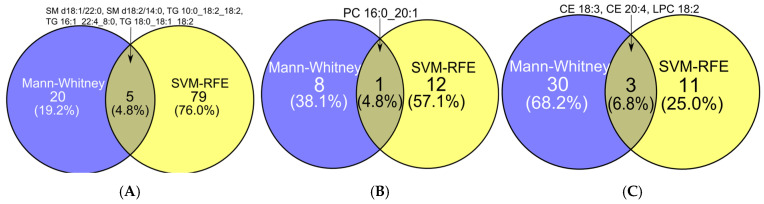
Comparative analysis (Venn diagrams) of biomarker sets identified by Mann–Whitney U test (blue) and SVM-RFE feature selection (yellow) methods in binary classification tasks: (**A**) Malignant versus benign tumor discrimination. (**B**) Benign tumor versus healthy control differentiation. (**C**) Malignant versus control classification.

When evaluating the overall performance of binary feature selection methods, both SVM-RFE and Mann–Whitney achieved the highest total score (23 points). However, SVM-RFE was preferred due to its superior stability. Despite their equal scores, the marker sets from these methods showed minimal overlap. For instance, only five lipids were common to both sets when distinguishing benign from malignant tumors: SM d18:1/22:0, SM d18:2/14:0, TG 10:0_18:2_18:2, TG 16:1_22:4_8:0, and TG 18:0_18:1_18:2 (Figure 1A). Similarly, PC 16:0_20:1 was the sole shared lipid between marker sets for controls versus benign tumors (Figure 1B), while CE 18:3, CE 20:4, and LPC 18:2 were common to both methods for controls versus malignant tumors (Figure 1C).

#### 2.3.2. Comparative Performance and Stability of Feature Selection Methods in Multiclass Analysis

The Kruskal–Wallis and Partial Least Squares Discriminant Analysis (PLS-DA) selection methods demonstrated the highest quality metrics, with the Kruskal–Wallis approach showing slightly greater stability in its feature set (Table 4). In contrast, RF and LASSO-based methods failed to produce stable feature sets across iterations (Table S2).

**Figure 2:**
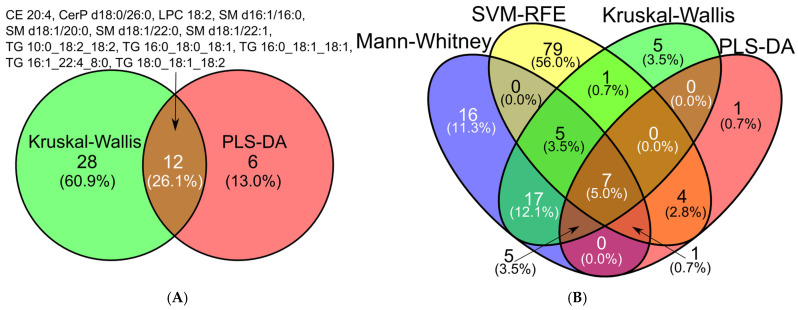
Comparative biomarker discovery in OC/benign tumor/control classification using multivariate feature selection approaches: (**A**) Kruskall–Wallis and PLS-DA; (**B**) Kruskall–Wallis, Mann–Whitney, PLS-DA and SVM-RFE.

A significant overlap was observed between the Kruskal–Wallis and PLS-DA-derived marker sets, with more than 25% of features shared between the two (Figure 2A). Notably, seven features were consistently identified across all multiclass and binary selection methods: SM d18:1/22:0, SM d18:1/22:1, TG 10:0_18:2_18:2, TG 16:1_22:4_8:0, TG 18:0_18:1_18:2, CE 20:4, and LPC 18:2 (Figure 2B, Table S3).

### 2.4. Machine Learning Models in Ovarian Tumor Classification

The machine learning models were optimized using Particle Swarm Optimization (PSO) to determine their ideal hyperparameters. This included three SVM configurations with distinct kernel functions (polynomial, radial basis function, and sigmoid), three neural network architectures (Multilayer Perceptron (MLP), CNN, and Residual Convolutional Neural Network (ResNet)), and an Extreme Gradient Boosting (XGBoost) model. The resulting optimal hyperparameter sets for each algorithm are comprehensively presented in Supplementary Table S4. For distinguishing between benign and malignant tumors, a CNN-based model utilizing the Mann–Whitney marker set achieved the highest performance, with accuracy and mean recall both at 0.81, along with 77% sensitivity and 84% specificity. In classifying benign tumors versus controls, an XGBoost model with SVM-RFE-selected features demonstrated exceptional results, achieving 0.96 accuracy and 0.95 mean recall, with perfect sensitivity (100%) and high specificity (90%). For malignant tumor versus control classification, both RF (SVM-RFE set) and XGBoost (Mann–Whitney set) models performed equally well, each showing 0.92 accuracy and mean recall, 93% sensitivity, and 90% specificity.

**Figure 3:**
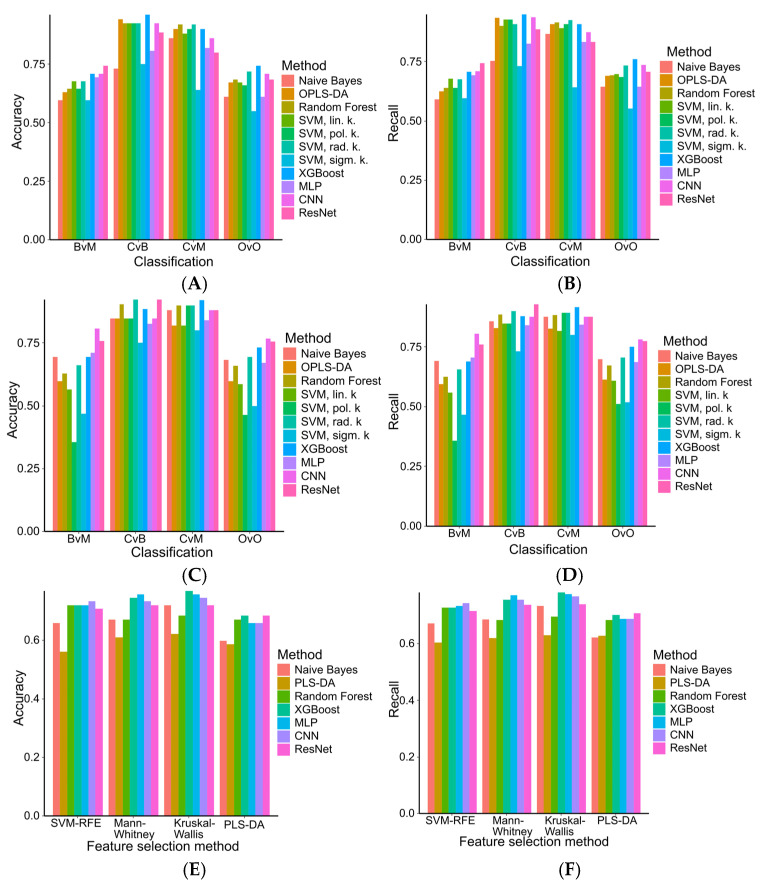
Performance evaluation (accuracy and mean recall) of machine learning models using different feature selection strategies: SVM-RFE binary feature selection (**A**,**B**), and Mann–Whitney feature selection (**C**,**D**) for binary classification and multiclass classification (**E**,**F**). BvM—benign versus malignant tumor separation, CvB—control versus benign group separation, CvM—control versus malignant group separation, OvO—one-versus-one classification.

**Figure 4:**
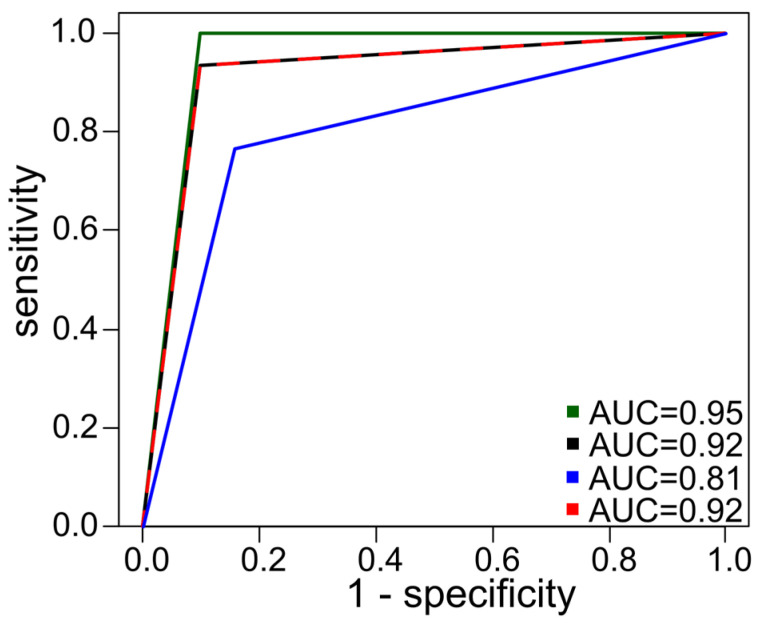
Receiver operating characteristic (ROC) analysis of optimal binary classification model for OC detection. AUC—area under the curve. Green color indicates XGBoost with SVM-RFE-selected features for control versus benign group separation; black represents RF with SVM-RFE features for control versus malignant group separation; blue corresponds to CNN with Mann–Whitney-selected features for benign versus malignant tumor separation; and red denotes XGBoost with Mann–Whitney features for control versus malignant group separation.

In one-versus-one (OvO) classification, the CNN model with Mann–Whitney markers yielded the best results (accuracy: 0.78, mean recall: 0.77) (Figure 3A–D and Figure 4, Tables S5 and S6). Overall, models using Mann–Whitney-selected features showed non-significantly higher accuracy (median: 0.78, IQR: 0.67–0.85) and mean recall (0.85, IQR: 0.76–0.90) compared to those using SVM-RFE features (accuracy: 0.74, IQR: 0.67–0.90; mean recall: 0.74, IQR: 0.68–0.90; *p* = 0.28 for accuracy, *p* = 0.17 for recall).

For multiclass classification, XGBoost with Kruskal–Wallis-selected features achieved the highest performance (accuracy: 0.78, mean recall: 0.77) (Figure 3E,F, Table S7). Models using Kruskal–Wallis markers consistently outperformed others, showing significantly higher accuracy (median 0.72, IQR 0.70–0.74) than PLS-DA-based models (median 0.66, IQR 0.63–0.67, *p* = 0.02) and marginally higher than SVM-RFE (median 0.72, IQR 0.69–0.72, *p* = 0.07) and Mann–Whitney (median 0.72, IQR 0.67–0.73, *p* = 0.05) sets. Similarly, they demonstrated significantly better mean recall (median 0.74, IQR 0.71–0.77) versus PLS-DA (median 0.68, IQR 0.63–0.69) and Mann–Whitney sets (median 0.74, IQR 0.68–0.75), in both cases *p* = 0.01 and a non-significant improvement over SVM-RFE (median 0.73, IQR 0.70–0.73), *p* = 0.10).

Comparing the top multiclass models, XGBoost (Kruskal–Wallis set) and the OvO CNN (Mann–Whitney set) showed comparable overall accuracy and recall. However, XGBoost had lower malignant tumor recall (60%) and benign tumor precision (69%), but higher benign tumor recall (84%) and malignant tumor precision (90%) relative to the CNN model (87%, 95%, 63%, 68%, respectively) (Table 5). Finally, XGBoost (median accuracy: 0.74, IQR: 0.72–0.89; recall: 0.76, IQR: 0.72–0.89) and CNN models (accuracy: 0.76, IQR: 0.73–0.85; recall: 0.77, IQR: 0.74–0.88) performed similarly (*p* = 0.21 for accuracy, *p* = 0.57 for recall), while CNNs significantly outperformed MLP in both metrics (median 0.74, IQR 0.69–0.81, *p* = 0.02 for accuracy, (median 0.75, IQR 0.69–0.83, *p* = 0.006 for recall).

## 3. Discussion

The identification of optimal feature sets represents a fundamental step in developing reliable predictive models for tumor classification, with significant implications for diagnostic accuracy and clinical decision-making [27]. Feature selection methods such as Mann–Whitney U tests and SVM-RFE employ distinct statistical approaches yet frequently generate models with similar overall performance metrics [37]. A closer examination reveals important distinctions between these approaches. Feature sets derived from Mann–Whitney testing demonstrate particularly strong performance in cluster separation quality, as quantified by established validation metrics. The elevated Davies–Bouldin index scores indicate more compact and well-separated clusters, while improved Hubert–Levin’s C index values reflect superior between-class discrimination. These properties suggest that Mann–Whitney selected features may be particularly valuable for applications requiring clear pathological categorization, such as distinguishing between benign and malignant tumor subtypes.

The age disparity between OC patients and both benign tumor and control groups introduces a potential confounding factor, given known age-related changes in blood lipid and metabolite profiles [38]. However, only 18 features showed a weak age association (0.30 >|r| > 0.20), while 87 had minimal correlation (|r| ≤ 0.20), confirming malignancy status as the primary determinant of the observed differences.

In Mann–Whitney-based panels, four age-associated lipids (DG 18:1_18:1, PC 16:0_18:3, TG 16:0_18:1_18:1, and TG 18:0_18:1_18:2) appeared in both panels discriminating malignant tumors from control and benign groups. Another four (PE 16:0_22:6, PS 16:0_20:3, LPC 18:2, and PE P-18:0/18:2) were specific to the malignant vs. control comparison. In contrast, only two lipids (PC 16:0_16:0 and TG 18:0_18:1_18:2) were shared in the malignant vs. benign panel, and just one (LPC 18:2) overlapped between both malignant tumor comparisons in SVM-RFE-based panels (Supplementary Figure S1). This suggests that SVM-RFE panels are more age-stable than Mann–Whitney panels.

Notably, LPC 18:2—which was included in an OPLS-DA panel distinguishing controls from benign tumors (groups without significant age differences)—has been linked to pancreatic [39] and colorectal [40] cancer in age-matched studies. Similarly, elevated blood triglycerides correlate with increased ovarian cancer risk in age-adjusted cohorts [41], and PC 16:0_16:0 shows malignancy-associated elevation independent of age [42,43].

The SVM-RFE approach demonstrates complementary strengths in predictive modeling applications. Lopez and colleagues provided compelling evidence that models built using SVM-RFE selected features surpass those utilizing RF or Relief-based selection in terms of classification accuracy and biomarker [44]. This advantage likely stems from SVM-RFE’s iterative optimization process, which evaluates feature importance within the context of the classifier’s decision boundary rather than relying solely on univariate statistical tests [27]. Such characteristics make SVM-RFE particularly effective for complex discrimination tasks where multiple biomarkers interact in non-linear ways to determine pathological status [45].

However, as Barbieri’s research team demonstrated, the performance of any feature selection method depends critically on dataset characteristics [46]. Factors including sample size, class imbalance, measurement noise, and biological heterogeneity all substantially influence which selection approach proves most effective. For instance, in datasets with strong effect sizes and minimal confounding variables, simpler univariate methods like Mann–Whitney may suffice [47]. Conversely, in scenarios involving high-dimensional data with numerous correlated features, more sophisticated techniques like SVM-RFE or embedded methods may be necessary to capture complex biomarker interactions [48].

The choice of feature selection methodology also carries important implications for model translation into clinical practice [49]. While computationally intensive methods may achieve marginally better performance in research settings, simpler approaches often prove more practical for clinical implementation due to easier validation and interpretation. This trade-off between performance and practicality underscores the need for careful method selection aligned with the specific application requirements and implementation constraints [50]. Future research directions should focus on developing adaptive selection frameworks that can automatically adjust to dataset characteristics while maintaining biological interpretability and clinical relevance.

This study represents a significant advancement in OC diagnostics by being the first to systematically identify optimal machine learning algorithms for analyzing complex multi-omics data (plasma metabolites and lipids) in a large clinical cohort of 229 OC patients. Our comprehensive evaluation demonstrates that XGBoost and RF models achieve an exceptional balanced accuracy of 92% for differentiation of OC from controls, with 93% sensitivity and 90% specificity—a performance level that compares favorably with established metabolomic approaches [51]. Notably, while Ban et al. found that SVM outperformed Adaptive Boosting and RF [51], our results underscore the critical importance of algorithm selection tailored to specific data characteristics and diagnostic objectives [52]. In classifying benign tumors versus controls, our XGBoost model with SVM-RFE-selected features achieved perfect sensitivity (100%) and high specificity (90%). Similarly, Fei Long et al. (2025) identified plasma extracellular vesicle metabolites as highly discriminative biomarkers, with SVM and RF models achieving an AUC of 0.94 in differentiating OC from benign tumors [11].

While plasma metabolites show great promise, protein-based markers remain clinically relevant, though their performance varies depending on analyte combinations and detection methods. Diagnostic panels incorporating fibrinogen, D-dimer, and the well-established CA-125 marker have achieved notable sensitivity (92%) and specificity (79%) in some studies [53]. More complex protein signatures, such as those combining CA125 with IGFBP2, SPP1, TSP1, and ADI, have demonstrated accuracy comparable to advanced XGBoost models [54,55,56]. Similarly, logistic regression models using IL-8 and TNFα [6], as well as neutrophil gelatinase-associated lipocalin/matrix metallopeptidase-9 complexes [5], have shown diagnostic performance on par with machine learning approaches. However, multi-analyte models exhibit considerable variability; for instance, combinations of CA125, CCL20, and menopausal status yielded reduced accuracy (77%) compared to lipidomic-based XGBoost models (81%) [7]. This underscores the necessity of rigorous biomarker selection and validation.

MLP architecture represents a fundamental yet powerful type of artificial neural network particularly well-suited for clinical research applications. Its versatility stems from the ability to directly process diverse data types including-omics profiles, categorical clinical variables, and continuous numerical measurements [57,58,59,60]. This inherent flexibility has established MLP as a widely adopted approach across various clinical prediction tasks. Most notably, MLPs operate effectively on properly scaled data without necessitating transformation into pseudo-continuous representations. This characteristic significantly reduces preprocessing complexity and minimizes potential error introduction during data conversion steps [33].

The performance of MLPs varies significantly depending on the nature of the classification task, the dataset characteristics, and the comparative machine learning models [61]. In our study, MLPs exhibited lower diagnostic accuracy in binary classification tasks but demonstrated superior performance in multiclass problems. This aligns with findings from Wang et al., where MLPs outperformed SVMs, suggesting that their hierarchical learning structure may be better suited for complex, multi-category discrimination [62].

However, the effectiveness of MLPs is not universally consistent across studies. For instance, Long et al. reported that MLPs were less accurate than both RF and SVM models, contrasting with our observation that MLPs surpass Naive Bayes (NB) classifiers [11]. This discrepancy may stem from differences in dataset composition, feature selection, or model hyperparameter tuning. Interestingly, in the differential diagnosis of inflammatory myopathy subtypes, MLPs ranked below RF and SVM but still exceeded the performance of NB, reinforcing the notion that MLPs occupy an intermediate position among machine learning classifiers in certain biomedical applications [14].

Notably, MLPs exhibit strong diagnostic capabilities in specific clinical contexts. For example, in Parkinson’s disease detection, MLPs and XGBoost models achieve high classification accuracy, whereas RF and SVM underperform [15]. This suggests that neural network-based approaches may be particularly effective for neurodegenerative disorder diagnostics, possibly due to their ability to capture non-linear patterns in heterogeneous biomedical data.

CNNs have emerged as powerful tools for diagnostic applications, consistently outperforming traditional machine learning methods across multiple studies [63,64,65,66,67]. However, CNN efficacy is highly dependent on dataset characteristics, with sample size being a critical limiting factor. Several studies have reported significant performance degradation when CNNs are applied to smaller datasets [68,69,70]. This data-hungry nature of deep learning architectures means that in resource-constrained scenarios with limited sample availability, simpler machine learning methods may offer comparable diagnostic accuracy while providing additional benefits in terms of computational efficiency and interpretability [33].

Notably, our findings indicate that CNNs consistently outperform MLPs, particularly when the input data can be effectively transformed into an image-like representation. This performance gap highlights the importance of proper data structuring for neural network applications. The process of converting conventional tabular data into artificial image formats, while computationally intensive, appears justified by the subsequent improvements in model accuracy [71].

This study advances OC diagnostics through a rigorous, large-scale integration of metabolomic and lipidomic data with machine learning. Our rigorous approach encompasses several strengths. First, the study leverages a substantial clinical cohort of 229 patients, including carefully matched comparison groups of benign ovarian neoplasms and healthy controls, enabling rigorous differential diagnosis evaluation. Second, the implementation of strict selection criteria—including sample collection prior to any therapeutic intervention or surgical procedure—ensures minimal confounding from treatment effects while providing a clear window into disease-specific metabolic alterations.

The usage of blood plasma as a biospecimen offers particular clinical advantages, being both minimally invasive and readily accessible for potential diagnostic implementation. Our deep metabolic profiling approach combines complementary analytical platforms: comprehensive lipidomic analysis using HPLC-MS in both ionization modes with MS/MS identification, coupled with NMR-based characterization of the low-molecular-weight metabolome. This dual-platform strategy provides exceptional coverage of both hydrophobic and hydrophilic metabolite fractions, capturing a more complete metabolic signature than single-platform approaches.

From a computational perspective, the study makes three key contributions: (1) an exhaustive systematic comparison of feature selection methods and classification algorithms, revealing context-dependent performance advantages; (2) a sophisticated evaluation of binary versus multiclass classification strategies, including OvO architecture benchmarking; and (3) implementation of PSO to efficiently explore over 400 hyperparameter combinations per method-task pairing, ensuring robust model configuration. The resulting models demonstrated high discriminatory power, with XGBoost achieving 96% accuracy in benign versus control classification and CNNs reaching 81% accuracy in malignant versus benign differentiation. Beyond diagnostic performance, these models provide valuable biological insights into OC metabolism through their identified feature signatures. Furthermore, the study establishes a methodological framework that could be extended to other cancers or multi-omics investigations.

While this study provides promising insights, several limitations should be acknowledged. First, although our cohort was substantial, the relatively small number of healthy controls may affect the generalizability of results. While this imbalance reflects real-world referral patterns in specialty centers, it underscores the need for cautious interpretation. To address this imbalance, we employed safe-level SMOTE to generate fifty synthetic control samples while carefully preserving data integrity. Importantly, we conducted feature selection prior to SMOTE application to minimize its impact on variable variability. This approach has proven effective in numerous clinical studies facing similar imbalance challenges [57,72,73,74,75], consistently improving model performance metrics including F-value and Youden index [76,77,78,79,80]. While SMOTE enhances minority class representation by increasing inter-sample correlations, it maintains the original sample structure without artificially altering internal data relationships [81]. Recent comparative analyses, including the work by Welvaars et al. (2023), demonstrate that while resampling methods like SMOTE improve classification performance, they may introduce overestimation of positive predictions [82]. This finding emphasizes the importance of carefully defining clinical prediction tasks when implementing such techniques. Our methodological approach, combining prudent feature selection with targeted SMOTE application, represents a balanced strategy for developing more robust clinical decision support tools while acknowledging these inherent limitations.

The single-center design presents another constraint. Despite robust internal validation, our models’ performance may not fully translate to broader populations due to variations in demographics, imaging protocols, and diagnostic workflows across institutions. While PSO successfully enhanced model discrimination by maximizing predictive performance, it cannot eliminate the risk of overfitting. The lack of external validation remains a critical concern, particularly given the variability observed in biomarker selection across different feature selection methods. These methodological challenges highlight the essential need for independent validation in diverse cohorts and verification through orthogonal analytical approaches.

From a translational perspective, while our machine learning models performed well, their clinical adoption faces challenges. Complex algorithms, particularly deep learning, often lack interpretability, which is critical for physician trust and regulatory approval. Moreover, we did not evaluate practical implementation barriers such as cost-effectiveness, assay reproducibility, or workflow integration—key factors for real-world utility.

Despite these limitations, our findings lay groundwork for developing liquid biopsy tests that could complement existing diagnostics. The identified metabolic signatures may provide new insights into OC pathogenesis and reveal novel therapeutic targets. To facilitate translation, we propose: (1) prospective multicenter validation with standardized protocols, (2) expanded cohorts to enhance statistical power, and (3) integration of multi-omics data to improve predictive accuracy and biological insight.

## 4. Materials and Methods

### 4.1. Study Design

The OC cohort comprised patients who underwent cytoreductive surgery at the V.I. Kulakov National Medical Research Center for Obstetrics, Gynecology, and Perinatology (NMRC for OGP, Moscow, Russia) between November 2019 and July 2020. The study included 229 participants divided into three distinct groups. The OC group consisted of 103 patients with histologically verified serous ovarian tumors, comprising 59 cases of high-grade serous carcinoma (including 10 at FIGO stages IA-IIB and 49 at stages IIC-IVA), 16 low-grade serous carcinomas, and 28 serous borderline tumors. For comparison, 107 patients with benign ovarian pathologies were enrolled, including 30 serous cystadenomas, 56 endometrioid cysts, and 21 mature teratomas. Additionally, 19 healthy women without any ovarian pathology formed the control group, with their status confirmed through comprehensive clinical evaluation involving detailed medical history, pelvic ultrasound examination, complete blood tests (both clinical and biochemical parameters), and assessment of specific tumor markers (CA125 and HE4) accompanied by ROMA and RMI calculations.

The study adhered to the ethical standards of the institutional research committee, Russian federal laws, and the 1964 Helsinki Declaration (and its later amendments). Written informed consent was obtained from all participants, and the study protocol (No. 10, 5 December 2019) was approved by the NMRC for OGP Ethics Committee.

Patients in the OC group were required to have histologically confirmed serous ovarian carcinoma within FIGO stages I-IV, while those in the comparison group needed histological verification of benign ovarian lesions (serous cystadenomas, endometrioid cysts, or mature teratomas).

Uniform exclusion criteria applied to all study groups included: (1) age < 18 years; (2) current or recent (≤6 months) hormonal therapy (oral contraceptives or hormone replacement therapy); (3) confirmed BRCA mutations; and (4) significant comorbidities including diabetes mellitus, active inflammatory/infectious diseases, or current pregnancy. The OC group had additional exclusions for patients with primary multiple malignancies or mixed epithelial ovarian tumor histologies. Both the benign lesion comparison group and healthy controls were excluded for any history of pelvic surgeries or prior malignancy diagnoses.

Blood samples were collected preoperatively in K2EDTA vacutainer tubes prior to administration of any perioperative medications (including antibiotics and analgesics). Whole blood was immediately processed by two-step centrifugation: first at 300× *g* for 20 min at 4 °C to separate cellular components, followed by collection of the supernatant which underwent secondary centrifugation at 12,000× *g* for 10 min at room temperature to obtain platelet-poor plasma. The final plasma aliquots were transferred to pre-labeled cryovials using wide-bore pipette tips to minimize shear stress, and immediately stored at −80 °C in a monitored freezer until analysis.

### 4.2. Lipidomic Analysis of Blood Plasma Samples (HPLC-MS)

Lipidomic profiling was conducted using an established laboratory protocol [13,83,84]. Plasma lipid extraction was performed via a modified Folch method where 40 μL of plasma was mixed with 480 μL of chloroform:methanol (2:1, *v*/*v*) and vortexed in an ultrasonic bath for 10 min. After adding 150 μL of deionized water, the mixture was centrifuged at 13,000× *g* for 5 min at 20 °C. The organic phase containing lipids was collected, evaporated under a gentle nitrogen stream, and reconstituted in 200 μL of isopropanol:acetonitrile (2:1, *v/v*).

To ensure analytical reliability, pooled quality control (QC) samples were prepared by combining equal 50 μL aliquots from all study participants’ samples, creating a representative reference matrix. Blank samples were prepared with isopropanol:acetonitrile (2:1, *v/v*) solvent mixture. QC samples were systematically injected every 10 study samples throughout the HPLC-MS batch runs. For each sample batch analysis, the first three samples analyzed were blanks, and before each QC samples blank sample was analyzed.

Chromatographic separation was achieved using an Ultimate 3000 HPLC system (Thermo Scientific, Bremen, Germany) coupled to a Maxis Impact qTOF mass spectrometer (Bruker Daltonics, Bremen, Germany). Separation was performed on a Zorbax XDB-C18 column (250 × 0.5 mm, 5 μm; Agilent, Santa Clara, CA, USA) maintained at 50 °C with a flow rate of 35 μL/min. The mobile phase consisted of Eluent A (10 mM ammonium formate with 0.1% formic acid in water:acetonitrile [40:60, *v/v*]) and Eluent B (10 mM ammonium formate with 0.1% formic acid in isopropanol:acetonitrile:water [90:8:2, *v/v/v*]). A linear gradient increased Eluent B from 30% to 95% over 25 min.

MS analysis of study samples was performed in both positive (400–1500 *m/z*) and negative (100–1000 *m/z*) ionization modes with capillary voltages of +4.1 kV and −3.0 kV, respectively. The nebulizer gas pressure was maintained at 0.7 bar with a dry gas flow of 6 L/min at 200 °C. For comprehensive lipid identification, data-dependent MS/MS acquisition was performed on QC samples. The instrument dynamically selected the top three most intense precursor ions from each full scan for fragmentation, applying a normalized collision energy of 35 eV. A dynamic exclusion window of 60 s was implemented to prevent repeated fragmentation of dominant ions, ensuring broader coverage of lower-abundance species.

Lipid identification was performed using LipidMatch [85] after data preprocessing, with inter-batch normalization by autoscaling [86]. All lipid species are reported according to LIPID MAPS classification [87].

### 4.3. Metabolomic Analysis by NMR Spectroscopy

Plasma metabolomic profiling was performed using 700 MHz NMR spectroscopy. Two phosphate buffer systems were prepared: Buffer A consisted of 80:20 H_2_O/D_2_O (*v*/*v*) sodium-phosphate buffer (pH 7.4) containing 6.15 mM sodium azide (NaN_3_) and 4.64 mM 3-(trimethylsilyl)propionic-2,2,3,3-d_4_ acid (TSP, Cambridge Isotope Laboratories Inc., Leicestershire, UK) sodium salt as an internal reference. Buffer B contained sodium-phosphate buffer in D_2_O (pH 7.4) with 1.5 M K_2_HPO_4_, 2 mM NaN_3_, and 4 mM TSP. For analysis, 120 μL of plasma was mixed with 120 μL of buffer solution, and 190 μL of this mixture was transferred to 5 mm NMR tubes (Bruker BioSpin Ltd., Ettlingen, Germany) and maintained at 6 °C until measurement.

All ^1^H-NMR spectra were acquired on a Bruker 700 MHz AVANCE NEO spectrometer (Bruker BioSpin, Ettlingen, Germany) equipped with a Prodigy cryogenic probe at 37 °C, with temperature calibration performed using d_4_-methanol (99.8% purity). The acquisition employed a Carr–Purcell–Meiboom–Gill (CPMG) pulse sequence with presaturation for water suppression, incorporating 128 refocusing pulses (0.6 ms echo delay each) for a total T_2_ filtering period of 78 ms. Following 4 dummy scans, spectra were collected with 73,728 data points across a 12,019 Hz spectral width.

Metabolite identification was performed using Bruker Biorefcode (Bruker BioSpin, Ettlingen, Germany) by matching both 1D and 2D J-resolved spectra against reference libraries. Semi-automated quantification was conducted using Chenomx NMR Suite 9.0 (Chenomx Inc., Edmonton, AB, Canada), with metabolite concentrations calculated relative to the 0.4 mM TSP reference standard [36].

### 4.4. Feature Selection and Stability Analysis

**Figure 5:**
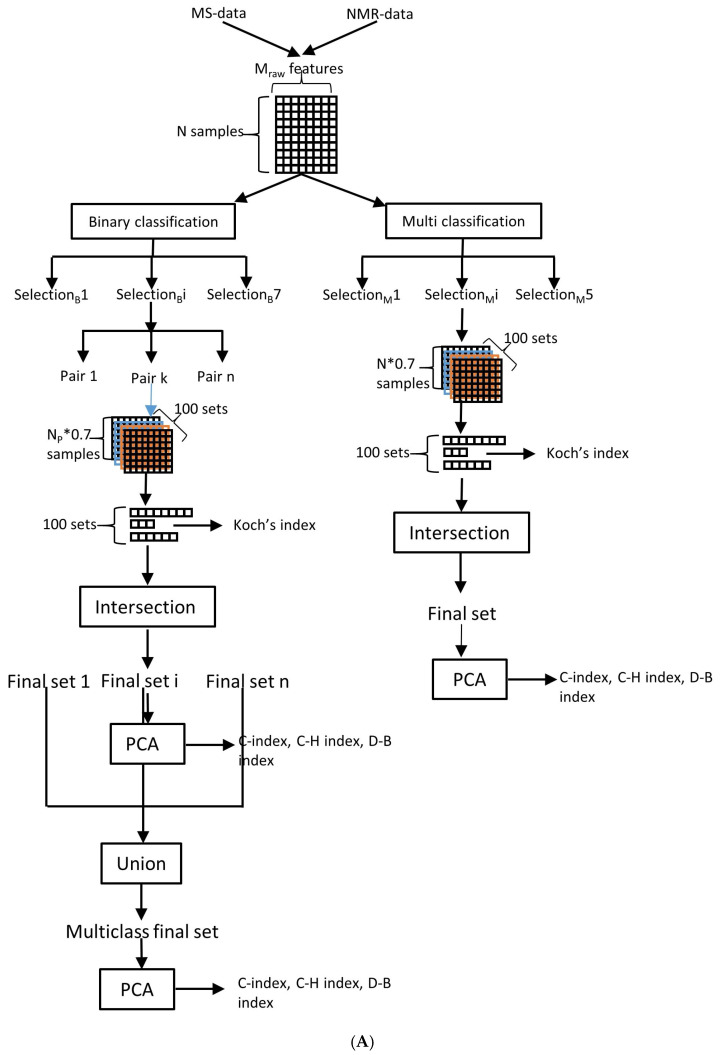

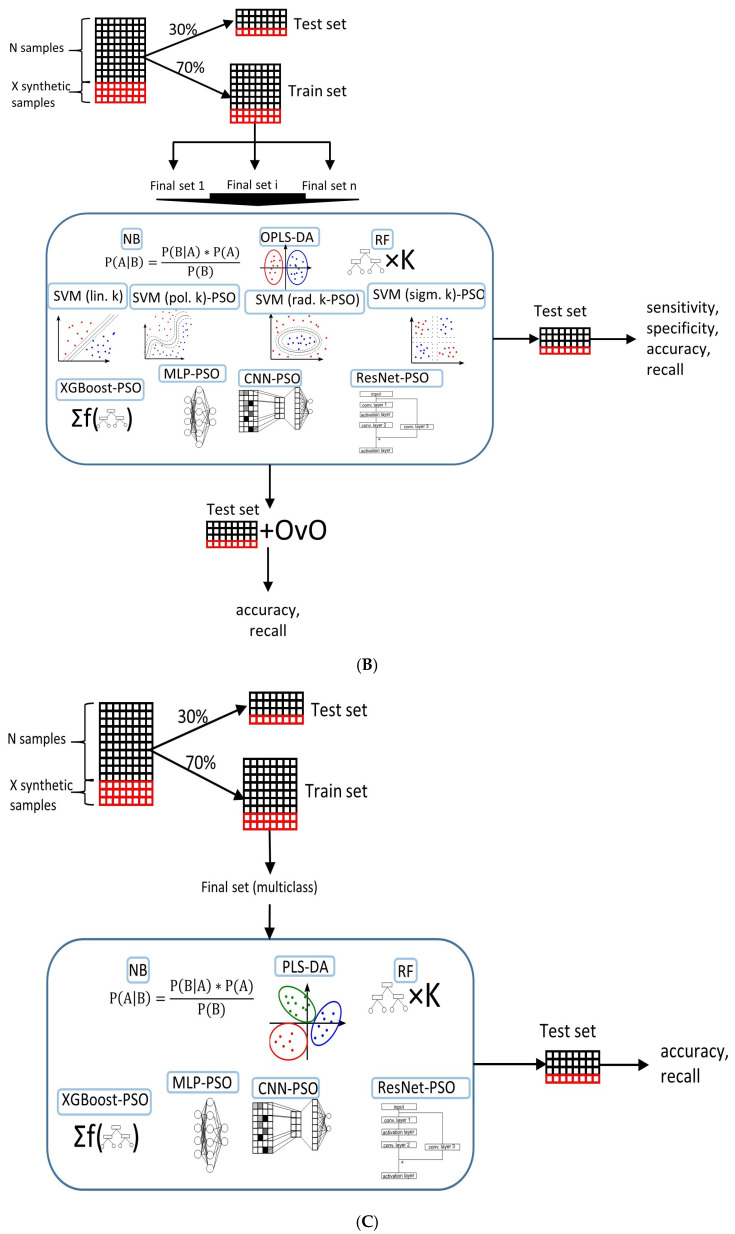
(**A**) Pipeline of feature selection methods. Selection_B_—methods for binary class selection features, selection_M_—methods for multiply feature selection. (**B**) Pipeline of binary classification methods. PSO—methods with particle swarm optimization tuning. (**C**) Pipeline of multiply classification methods. PSO—methods with particle swarm optimization tuning.

Lipidomic and metabolomic data were integrated and processed to reduce feature dimensionality. For binary classification, seven feature selection methods were employed: (1) Wilcoxon–Mann–Whitney test (*p* < 0.05), (2) Welch’s *t*-test (*p* < 0.05), (3) OPLS-DA with VIP > 1 [88], (4) RF (top √n features by Gini index) [89], SVM-RFE based on SVM weights (iterative elimination until model accuracy decreases) [90], LASSO (non-zero coefficients) [91], and (7) Boruta (all relevant selection) [92]. For multiclass classification, five methods were used: (1) Kruskal–Wallis test (*p* < 0.05), (2) PLS-DA, VIP > 1 (3) RF (top √n features) [89], (4) LASSO (non-zero coefficients) [91], and (5) Boruta (all relevant selection) [92] (Figure 5A).

Method stability was assessed through 100 iterations on randomly selected 70% subsamples. Features consistently selected in all iterations were considered robust group discriminators. The robustness of the feature selection method was further quantified using Koch’s biotic diversity index [93]. A PC space was constructed from the final selected features, and three cluster validation metrics were computed: (1) Hubert–Levin’s normalized C-index [94], (2) Davies–Bouldin index [95], and (3) Calinski–Harabasz pseudo-F statistic [96] (Figure 5A). Missing values were imputed as 100 (C-index/Davies–Bouldin) or −100 (Calinski–Harabasz).

Each method was ranked (7/5 to 1 for binary/multiclass) across all metrics, with the highest-scoring method’s features advancing to model selection.

### 4.5. Classification Model Selection

To address the significant class imbalance (1:5.6:5.4 ratio) in our dataset, we employed the safe-level Synthetic Minority Over-sampling Technique (SMOTE) [76], generating 50 synthetic control samples to improve model training [76]. The balanced dataset was then partitioned into training (70%) and test (30%) sets while preserving the original distribution patterns. For binary classification, eleven methods were evaluated: NB, OPLS-DA, RFt, SVM (with linear, polynomial, radial, and sigmoid kernels), XGBoost, MLP, CNN, and ResNet. Multiclass classification was performed using seven selected methods: NB, PLS-DA, RF, XGBoost, MLP, CNN, and ResNet (Figure 5B,C). Additionally, we implemented a OvO strategy for binary classifiers, where classification accuracy scores from individual binary models were aggregated to enhance multiclass prediction performance.

For CNN and ResNet models, input data were transformed into 2D representations using the DeepInsight methodology [97]. These models incorporated a GELU activation layer followed by dropout (rate = 0.1). All neural networks were trained with an initial learning rate of 0.01 and Adamax optimization; the learning rate decayed at 0.5 for all models except the binary ResNet, which used a decay rate of 0.9. Hyperparameter tuning for SVMs, XGBoost, and the architectures of MLP, CNN, and ResNet was performed via PSO [98].

All analyses were conducted in R 4.3.3 using the following packages: ropls [99], RandomForest [100], e1071 [101], glmnet [102], boruta [92], xgboost [103], clustersim [104], smotefamily [105], lsa [106], tsne [107], cxhull [108], caret [109], keras [110].

## 5. Conclusions

The integration of multi-omics data with advanced machine learning algorithms represents a transformative approach in OC diagnostics. Our large-scale analysis of plasma metabolites and lipids systematically evaluated different feature selection strategies and machine learning models for tumor classification, comparing their performance in both binary and multiclass settings. Among binary classification approaches, SVM-RFE and Mann–Whitney methods demonstrated comparable performance scores. However, SVM-RFE emerged as the preferred choice due to its significantly higher stability (mean stability score: 0.75 vs. 0.40), despite limited biomarker overlap between the two methods.

For multiclass classification, Kruskal–Wallis and PLS-DA-based selection methods demonstrated equivalent performance metrics, with Kruskal–Wallis showing slightly superior feature selection stability (0.47 compared to 0.46). These methods exhibited substantial feature overlap, sharing more than 25% common markers while identifying seven consensus biomarkers across all selection approaches.

The machine learning model analysis yielded several critical findings. CNN architectures utilizing Mann–Whitney selected features achieved optimal performance in malignant versus benign classification, attaining 81% accuracy and mean recall. XGBoost models utilizing SVM-RFE features excelled in benign versus control classification, achieving exceptional 95% accuracy with perfect 100% sensitivity. In multiclass evaluation, XGBoost models incorporating Kruskal–Wallis selected features reached the highest classification accuracy of 78%, representing statistically significant improvement over alternative methods.

The clinical implementation of these advanced diagnostic models will require careful consideration of practical factors, including assay standardization, reproducibility across platforms, and integration with existing clinical workflows. Nevertheless, the demonstrated performance of machine learning-driven multi-omics analysis offers a promising path toward more accurate, earlier, and potentially more accessible OC detection, addressing a critical unmet need in women’s health care.

From a translational perspective, these findings open new avenues for developing liquid biopsy tests that could complement or even reduce reliance on current diagnostic methods. The identification of robust metabolic and lipidomic signatures through machine learning approaches may also provide insights into OC pathogenesis and reveal new therapeutic targets. As the field progresses, continued refinement of these models through larger multicenter studies and the incorporation of additional -omics layers (such as proteomics and transcriptomics) may further enhance their diagnostic and prognostic utility.

## Figures and Tables

**Table 1:** Clinical characteristics of study participants.

Variable	OC (*n* = 103)	Benign Tumor (*n* = 107)	Control Group (*n* = 19)	*p* Value (Kruskal–Wallis H Test)
Age, years, Median (Q1;Q3)	51.0 (39.0;60.0)	38.0 (34.0;45.0)	39.5 (34.0;60.3)	<0.001
BMI (kg/m^2^), Median (Q1;Q3)	25.0 (22.0;27.8)	23.5 (21.0;27.0)	22.5 (20.8;25.3)	0.27
Benign ovarian tumors, *n* (%)	-	cystadenoma—30 (28%) endometrioid cyst—56 (52%) mature teratoma—21(20%)	-	-
Borderline tumors, *n* (%)	28 (27%)	-	-	-
Low-grade OC, *n* (%)	16 (16%)	-	-	-
FIGO stage (high-grade OC), *n* (%)	IA—5( 4.9%) IIB—5 (4.9%) IIC—1 (1.0%) IIIA—4 (3.9%) IIIC—40 (39%) IVA—4 (3.9%)	-	-	

**Table 2:** Comparative stability analysis of feature selection methods using Koch’s biotic diversity index for binary classification tasks. OPLS-DA—Orthogonal Projection on Latent Structures-Discriminant Analysis; RF—Random Forest; SVM-RFE—Support Vector Machine-Recursive Feature Elimination; LASSO—least absolute shrinkage and selection operator.

Method	Benign vs. Malignant	Control vs. Benign	Control vs. Malignant	Mean (Score)
Mann–Whitney	0.41	0.31	0.47	0.40 (5)
Welch	0.35	0.27	0.35	0.32 (4)
OPLS-DA	0.50	0.51	0.57	0.53 (6)
RF	0.19	0.20	0.16	0.18 (3)
SVM-RFE	0.94	0.59	0.71	0.75 (7)
LASSO	0.20	0.14	0.06	0.14 (1)
Boruta	0.17	0.14	0.12	0.14 (2)

**Table 3:** Cluster separation metrics in PC space for clinical groups using selected features.

Metric	Method	Benign vs. Malignant	Control vs. Benign	Control vs. Malignant	Combined Feature Set	Mean (Score)
Hubert–Levin’s C index	Mann–Whitney	0.46	0.42	0.45	0.44	0.44 (7)
	Welch	0.46	0.42	0.46	0.44	0.44 (6)
	OPLS-DA	0.45	0.46	0.47	0.47	0.46 (4)
	RF	100.00	100.00	100.00	0.47	75.12 (1)
	SVM-RFE	0.47	0.44	0.46	0.45	0.45 (5)
	LASSO	0.46	100.00	100.00	0.46	50.23 (2)
	Boruta	0.44	100.00	100.00	0.44	50.22 (3)
Davies–Bouldin’s index	Mann–Whitney	3.35	4.09	3.87	3.95	3.81 (7)
	Welch	3.33	4.37	4.46	4.49	4.16 (6)
	OPLS-DA	4.76	16.44	15.23	13.39	12.45 (4)
	RF	100.00	100.00	100.00	7.55	76.89 (1)
	SVM-RFE	10.75	7.43	7.66	8.77	8.65 (5)
	LASSO	2.80	100.00	100.00	2.80	51.40 (3)
	Boruta	2.72	100.00	100.00	2.72	51.36 (2)
Calinski–Harabasz pseudo-F statistic	Mann–Whitney	−12.79	47.35	−4.53	1.53	7.89 (4)
	Welch	−13.42	45.04	1.10	7.99	10.18 (5)
	OPLS-DA	4.25	30.06	24.15	23.39	20.46 (7)
	RF	−100.00	−100.00	−100.00	15.92	−71.02 (1)
	SVM-RFE	11.66	11.86	20.36	10.89	13.69 (6)
	LASSO	−14.29	−100.00	−100.00	−14.29	−57.14 (2)
	Boruta	8.82	−100.00	−100.00	8.82	−45.59 (3)

**Table 4:** Robustness and discriminatory power assessment of selected biomarker panels in multiclass OC classification.

Method	Koch’s Index (Score)	Hubert–Levin’s C Index (Score)	Davies–Bouldin’s Index (Score)	Calinski–Harabasz Pseudo-F Statistic (Score)	Sum Score (Rank)
Kruskall–Wallis	0.47 (5)	0.44 (4)	3.95 (5)	1.37 (4)	18 (1)
PLS-DA	0.46 (4)	0.42 (5)	7.69 (4)	15.19 (5)	18 (1)
RF	0.18 (1)	100.00 (3)	100.00 (3)	−100.00 (3)	10 (2)
LASSO	0.21 (3)	100.00 (2)	100.00 (2)	−100.00 (2)	9 (3)
Boruta	0.18 (2)	100.00 (1)	100.00 (1)	−100.00 (1)	5 (4)

**Table 5:** Prognostic performance of the best combinations of classification model and feature selection method across clinical groups.

Model, Feature Selection Method	Predicted Outcome	Clinical Group		
		Control (*n* = 20)	Benign (*n* = 32)	Malignant (*n* = 30)
XGBoost, Kruskal–Wallis set	control	18 (90%)	3	2
	benign	2	27 (84%)	10
	malignant	0	2	18 (60%)
OvO CNN, Mann–Whitney set	control	17 (85%)	5	3
	benign	0	20 (63%)	1
	malignant	3	7	26 (87%)

## Data Availability

Data are contained within the Supplementary Materials.

## Supplementary Materials

The supporting information can be downloaded at: https://www.mdpi.com/article/10.3390/ijms26146630/s1.

## Abbreviations

The following abbreviations are used in this manuscript:

ANN	Artificial neural network.
CE	Cholesterol esters
Cer(P)	Ceramide (phosphate)
CNN	Convolutional Neural Network
DG	Diacylglycerols
FIGO	International Federation of Gynecology and Obstetrics
LASSO	Least Absolute Shrinkage and Selection operator
MLP	Multilayer Perceptron
NB	Naive Bayes
OPLS-DA	Orthogonal Projection on Latent Structures-Discriminant Analysis
PLS-DA	Partial Least Squares Discriminant Analysis
PC (P-/O-)	(plasmenyl-/plasmanyl) Phosphatidylcholine
PE (P-/O-)	(plasmenyl-/plasmanyl) Phosphatidylethanolamine
PSO	Particle Swarm Optimization
ResNet	Residual Convolutional Neural Network
RFE	Recursive Feature Elimination
RF	Random Forest
SM	Sphingomyelins
SVM	Support Vector Machine
TG	Triacylglycerol
XGBoost	Extreme Gradient Boosting
VIP	Variable Importance projection
