# Supplementary material for: Machine Learning Framework for Ovarian Cancer Diagnostics Using Plasma Lipidomics and Metabolomics
Source: Int J Mol Sci. 2025 Jul 10;26(14):6630. doi: 10.3390/ijms26146630 (PMC12294939; doi:10.3390/ijms26146630)
Supplement: Supplementary file 1 [file ijms-26-06630-s001.zip › ijms-3698154-supplementary.pdf]

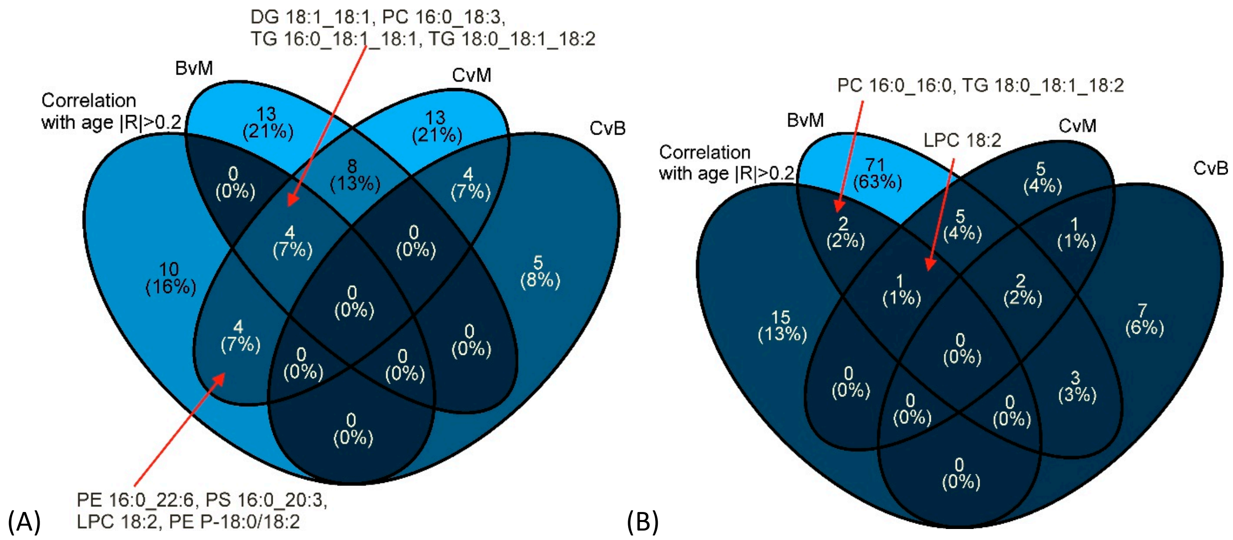

Figure S1. Overlapping biomarker panels with age-associated features (|r| > 0.2). BvM denotes panels discriminating plasma samples from patients with benign versus malignant tumors, CvM represents control group versus malignant tumor comparisons, and CvB indicates control group versus benign tumor discrimination. (A) Mann-Whitney-based panels; (B) SVM-RFE-based panels.

Table S1. Potential markers, selected by each feature selection method in three binary comparisons.

Method	Benign vs malignant	Control vs benign	Control vs malignant
Mann-Whitney	CerP d18:0/26:0, DG 18:1_18:1, PC 16:0_18:3, PC 18:2_22:6, PC O-20:0/18:1, PC O-20:0/18:2, PC P-20:0/18:1, PC P-22:1/18:2, OxPC(18:1_16:1 OH), SM d14:0/26:0, SM d16:1/14:0, SM d16:1/16:0, SM d18:1/20:0, SM d18:1/22:0, SM d18:1/22:1, SM d18:2/14:0, SM d22:0/18:2, TG 10:0_16:1_22:6, TG 10:0_18:0_20:4, TG 10:0_18:2_18:2, TG 16:0_18:0_18:1, TG 16:0_18:1_18:1, TG 16:1_22:4_8:0, TG 18:0_18:1_18:2, TG 18:0_18:2_20:3	CE 18:3, DG 18:1_18:2, PC 14:0_18:2, PC 16:0_20:1, PE P-16:0/20:4, PE P-18:0/18:2, Alanine, Acetate/Acetoacetate, n3-hydroxybutirate/acetoacetate	CE 18:3, CE 20:4, DG 16:0_18:1, DG 18:1_18:1, DG 18:1_18:2, LPC 18:1, LPC 18:2, LPC 20:3, PC 14:0_18:2, PC 16:0_18:3, PC 16:1_18:4, PC 18:2_22:6, PC 20:4_22:6, PE 16:0_22:6, PC P-16:0/18:2, PE P-18:0/18:2, PS 16:0_20:3, OxPC 18:1_16:1(COOH), SM d16:1/14:0, SM d18:1/20:0, SM d18:1/22:0, SM d18:2/14:0, SM d22:0/18:2, TG 10:0_16:1_22:6, TG 10:0_18:2_18:2, TG 16:0_18:0_18:1, TG 16:0_18:1_18:1, TG 16:1_18:0_18:1, TG 18:0_18:1_18:1, TG 18:0_18:1_18:2, Alanine, Glycine, Methanol
Welch	CE 22:6, CerP d18:0/26:0, OxPC 16:1_18:2 (OOO), OxPC 18:1_16:1 (OH), PC 16:0_18:3, PC 18:2_22:6, PC O-20:0/18:1, PC O-20:0/18:2, PC P-20:0/18:1, SM d14:0/26:0, SM d18:1/20:0, SM d18:1/22:0, SM d18:1/22:1, SM d18:2/14:0, SM d22:0/18:2, TG 10:0_16:1_22:6, TG 10:0_18:0_20:4, TG 10:0_18:2_18:2, TG	CE 18:3, DG 18:1_18:2, PC 14:0_18:2, PC 16:0_20:2, PC 16:0_20:1, PE 16:0_22:6, PC P-16:0/22:6, PS 16:0_20:3, Glutamine/Glucose, n3-	CE 18:1, CE 18:3, CE 20:4, DG 16:0_18:1, DG 18:1_18:1, DG 18:1_18:2, LPC 18:1, LPC 18:2, MGDG 18:0_20:1, OxPE 16:0_18:2(OOO), PC 14:0_18:2, PC 16:0_18:3, PC 16:1_18:4, PC 18:2_22:6, PE 16:0_22:6, PE P-18:0/18:2, PC O-18:0/18:1, PS 16:0_20:3, SM d16:1/14:0, SM d18:1/18:0, SM d18:1/20:0, SM

	16:0_18:0_18:1, TG 16:0_18:1_18:1, TG 16:1_22:4_8:0, TG 18:0_18:1_18:1, TG 18:0_18:1_18:2, TG 18:0_18:2_20:3	hydorxybutirate/AcO	d18:1/22:0, SM d18:1/22:1, SM d18:2/14:0, SM d20:0/16:1, TG 10:0_16:1_22:6, TG 10:0_18:2_18:2, TG 16:0_18:0_18:1, TG 16:0_18:1_18:1, TG 16:0_18:2_18:2, TG 16:1_18:0_18:1, TG 16:1_18:1_18:1, TG 18:0_18:1_18:1, TG 18:0_18:1_18:2, Glutamine/Valine, n3-Hydroxybutirate/acetate
OPLS-DA	CE 20:4, LPC 18:0, LPC 18:2, PC 16:0_22:6, SM d18:1/22:0, SM d18:1/22:1, SM d18:1/24:0, TG 10:0_18:2_18:2, TG 16:0_16:0_18:1, TG 16:0_18:1_18:1, TG 16:1_22:4_8:0	LPC 18:2, PC 16:0_20:3, PC 16:0_20:4, PC 16:0_22:6, PC 16:0_20:1, PC 18:0_18:2, PC 18:0_20:4, PC 18:0_20:5	CE 18:3, LPC 16:0, LPC 18:0, , MGDG 18:0_18:0, OxPC 20:4_14:0 (COOH), LPC 18:2, LPC 20:5, PC 14:0_18:2, PC 16:0_18:2, PC 16:0_20:2, PC 16:0_20:3, PC 16:0_20:4, PC 16:1_18:2, PC 16:0_20:1, PC 18:0_18:1, PC 18:0_18:2, PC 18:0_20:5, SM d18:1/18:0, SM d18:1/22:0, TG 10:0_18:2_18:2, TG 16:0_18:1_18:1, TG 16:0_18:1_18:2
Random Forest	PC P-20:0/18:1	-	DG 18:1_18:2
SVM-REF	CE 18:0, CE 18:3, CE 22:6, Cer-NS d18:1/22:0, Cer-NS d18:1/24:0, LPC 16:0, LPC 16:1, LPC 18:0, LPC 18:0, LPC 18:2, LPC 20:5, MGDG 16:0_20:2, MGDG 18:0_20:0, OxPC 18:0_18:4 (COOH), OxPC 18:0_20:5 (Ke, OH), OxPC 20:4_14:0 (COOH), OxPE 22:6_18:2 (OOO), OxPG 18:0_18:1 (1O), OxPG 18:1_18:0 (1O), OxPI 16:0_18:1 (1O), PA 20:0_20:1, PC 14:0_18:2, PC 14:0_22:6, PC 16:0_16:0, PC 16:0_16:1, PC 16:0_18:0, PC 16:0_18:2, PC 16:0_20:2, PC 16:0_20:4, PC 16:0_22:5, PC 16:0_22:6, PC 16:1_22:6, PC 16:0_20:1, PC 18:0_20:4, PC 16:0_20:5, PC 18:0_18:1, PC 18:0_18:2, PC 18:0_20:3, PC 18:0_22:4, PC 18:0_22:6, PC 18:2_20:3, PC 18:2_22:6, PC 20:3_22:3, PE 18:1_20:3, PE 18:0_20:5, PI 16:0_18:2, PI 16:0_20:3, PI 18:0_18:2, PC O-18:0/22:6, PC O-16:0/20:4, PC O-18:1/16:0, PC O-24:0/20:5, PC P-16:0/16:0, PC P-16:0/18:1, PC P-	CE 18:2, CE 20:4, PC 16:0_20:5, PC 16:0_22:6, PC 16:0_20:1, PC 18:0_22:6, PC P-20:0/20:4, TG 16:0_18:1_22:5, TG 16:0_18:2_18:2, TG 18:0_18:1_18:1, n3-Hydroxybutyrate, Acetone, Lactate	CE 18:1, CE 18:3, CE 20:4, LPC 18:2, MGDG 16:0_20:2, PC 16:0_20:5, PC 16:0_22:5, PC 16:0_20:1, PC 18:0_18:1, SM d18:1/22:1, SM d18:1/24:0, TG 12:0_18:2_18:3, PC 16:0_18:1, PC 18:0_18:2

	18:0/18:1, PC P-18:0/18:2, PC P-18:0/20:4, PC P-22:1/18:2, PE P-16:0/22:6, SM d18:1/20:0, SM d18:1/20:1, SM d18:1/22:0, SM d18:1/24:1, SM d18:2/14:0, SM d18:2/16:0, SM d22:0/18:1, SM d22:0/20:2, TG 10:0_18:2_18:2, TG 12:0_16:1_18:2, TG 16:0_16:0_18:1, TG 16:0_16:1_18:1, TG 16:0_18:1_22:5, TG 16:1_16:1_18:2, TG 16:1_22:4_8:0, TG 18:0_18:1_18:2, TG 18:1_18:1_18:2, TG 18:1_18:1_22:6, TG 18:1_18:2_18:2, TG 18:1_18:2_18:3, TG 18:2_18:2_18:3, Glutamate, Glutamine, Glucose/Citrate, Glucose/Pyruvate		
LASSO	SM d18:1/22:1, TG 16:1_22:4_8:0, TG 18:0_18:1_18:2, TG 18:0_18:2_20:3, PC P-20:0/18:1	-	-
Boruta	OxPC 18:1_16:1 OH), PC P-20:0/18:1	-	-

Table S2. Potential markers, selected by each feature selection method in multiclass comparisons.

Method	Marker
Kruskal-Wallis	CE 18:3, CE 20:4, CerP d18:0/26:0, DG 16:0_18:1, DG 18:1_18:1, DG 18:1_18:2, LPC 18:2, OxPC 18:1_16:1(OH), PC 14:0_18:2, PC 16:0_18:3, PC 16:1_18:4, PC 16:0_20:1, PC 18:2_22:6, PE 16:0_22:6, PC O-18:0/18:1, PC P-16:0/18:2, PC P-20:0/18:1, PE P-18:0/18:2, PS 16:0_20:3, SM d14:0/26:0, SM d16:1/14:0, SM d16:1/16:0, SM d18:1/20:0, SM d18:1/22:0, SM d18:1/22:1, SM d18:2/14:0, SM d20:0/18:1, SM d22:0/18:1, SM d22:0/18:2, TG 10:0_16:1_22:6, TG 10:0_18:0_20:4, TG 10:0_18:2_18:2, TG 16:0_18:0_18:1, TG 16:0_18:1_18:1, TG 16:1_18:0_18:1, TG 16:1_22:4_8:0, TG 18:0_18:1_18:2, TG 18:0_18:2_20:3, Alanine, Methanol
PLS-DA	CE 20:4, CerP d18:0/26:0, LPC 16:0, LPC 18:2, PC 16:0_20:4, SM d16:1/16:0, SM d18:1/20:0, SM d18:1/22:0, SM d18:1/22:1, SM d18:1/24:0, TG 10:0_18:2_18:2, TG 16:0_16:0_18:1, TG 16:0_18:0_18:1, TG 16:0_18:1_18:1, TG 16:1_22:4_8:0, TG 18:0_18:1_18:1, TG 18:0_18:1_18:2, Glucose
Random Forest	-
LASSO	-
Boruta	PC P-20:0/18:1

Table S3. Molecular features, common for each combination of sets, selected by feature selection methods in multiclass tasks.

Feature selection method	Features	Size
Mann-Whitney, SVM-REF, Kruskal-Wallis, PLS-DA	SM d18:1/22:0, SM d18:1/22:1, TG 10:0_18:2_18:2, TG 16:1_22:4_8:0, TG 18:0_18:1_18:2, CE 20:4, LPC 18:2	7

Mann-Whitney, Kruskal-Wallis, PLS-DA	CerP d18:0/26:0, SM d16:1/16:0, SM d18:1/20:0, TG 16:0_18:0_18:1, TG 16:0_18:1_18:1	5
Mann-Whitney, SVM-REF, PLS-DA	TG 18:0_18:1_18:1	1
Mann-Whitney, SVM-REF, Kruskal-Wallis	SM d18:2/14:0, CE 18:3, PC 14:0_18:2, PC 16:0_20:1, PC 18:2_22:6	5
SVM-REF, Kruskal-Wallis	SM d22:0/18:1	1
Mann-Whitney, Kruskal-Wallis	DG 18:1_18:1, PC 16:0_18:3, SM d14:0/26:0, SM d16:1/14:0, SM d22:0/18:2, TG 10:0_16:1_22:6, TG 10:0_18:0_20:4, TG 18:0_18:2_20:3, DG 18:1_18:2, Alanine, DG 16:0_18:1, PC 16:1_18:4, PE 16:0_22:6, PE P-18:0/18:2, PS 16:0_20:3, TG 16:1_18:0_18:1, Methanol	17
SVM-REF, PLS-DA	LPC 16:0, PC 16:0_20:4, TG 16:0_16:0_18:1, SM d18:1/24:0	4
Mann-Whitney	PC 18:2_22:6, PC O-20:0/18:1, PC O-20:0/18:2, PC P-20:0/18:1, PC P-22:1/18:2, OxPC(18:1_16:1 OH), PE P-16:0/20:4, PE P-18:0/18:2, Acetate/Acetoacetate, n3-hydroxybutirate/acetoacetate, LPC 18:1, LPC 20:3, PC 20:4_22:6, PC P-16:0/18:2, OxPC 18:1_16:1(COOH), Glycine	16
SVM-REF	CE 18:0, CE 22:6, Cer-NS d18:1/22:0, Cer-NS d18:1/24:0, LPC 16:1, LPC 18:0, LPC 20:5, MGDG 16:0_20:2, MGDG 18:0_20:0, OxPC 18:0_18:4 (COOH), OxPC 18:0_20:5 (Ke, OH), OxPC 20:4_14:0 (COOH), OxPE 22:6_18:2 (OOO), OxPG 18:0_18:1 (1O), OxPG 18:1_18:0 (1O), OxPI 16:0_18:1 (1O), PA 20:0_20:1, PC 14:0_22:6, PC 16:0_16:0, PC 16:0_16:1, PC 16:0_18:0, PC 16:0_18:2, PC 16:0_20:2, PC 16:0_22:5, PC 16:0_22:6, PC 16:1_22:6, PC 18:0_20:4, PC 16:0_20:5, PC 18:0_18:1, PC 18:0_18:2, PC 18:0_20:3, PC 18:0_22:4, PC 18:0_22:6, PC 18:2_20:3, PC 20:3_22:3, PE 18:1_20:3, PE 18:0_20:5, PI 16:0_18:2, PI 16:0_20:3, PI 18:0_18:2, PC O-18:0/22:6, PC O-16:0/20:4, PC O-18:1/16:0, PC O-24:0/20:5, PC P-16:0/16:0, PC P-16:0/18:1, PC P-18:0/18:1, PC P-18:0/18:2, PC P-18:0/20:4, PC P-22:1/18:2, PE P-16:0/22:6, SM d18:1/20:0, SM d18:1/20:1, SM d18:1/24:1, SM d18:2/16:0, SM d22:0/20:2, TG 12:0_16:1_18:2, TG 16:0_16:1_18:1, TG 16:0_18:1_22:5, TG 16:1_16:1_18:2, TG 18:1_18:1_18:2, TG 18:1_18:1_22:6, TG 18:1_18:2_18:2, TG 18:1_18:2_18:3, TG 18:2_18:2_18:3, Glutamate, Glutamine, Glucose/Citrate, Glucose/Pyruvate, CE 18:2, PC P-20:0/20:4, TG 16:0_18:2_18:2, n3-Hydroxybutyrate, Acetone, Lactate, CE 18:1, TG 12:0_18:2_18:3, PC 16:0_18:1	79
Kruskal-Wallis	OxPC 18:1_16:1(OH), PC O-18:0/18:1, PC P-16:0/18:2, PC P-20:0/18:1, SM d20:0/18:1	5
PLS-DA	Glucose	1

Table S4. The hyperparameters of the classification models for each set of markers, optimized by PSO. The kernel types include "pol" (polynomial kernel), "rad" (radial kernel), and "sigm" (sigmoid kernel). For the MLP, the key hyperparameter is h. layers, denoting the number of hidden layers. In the CNN, c. layers refers to the number of convolutional layers, while k. size indicates the size of convolutional kernels in each corresponding layer. Additionally, c. kernels specifies the number of convolutional kernels in a standalone convolutional layer, and c. k. size defines the size of those kernels. For the ResNet architecture, blocks represents the number of residual blocks. Within each residual block, b. kernels indicates the number of convolutional kernels per layer, and b. k. size denotes the size of the convolutional kernels in each layer of the block.

	Benign vs malignant	Benign vs control	Malignant vs control	Union binary markers	Multiclass markers
	SVM-REF				Kruskal-Wallis
SVM pol. k.	degree = 1.05, $\gamma=4.9*10^{-3}$, $coef_0=-66$	degree = 1.2, γ = 8.9, $coef_0$ = -100	degree = 2.8, $\gamma = 1.2*10^{-4}$, $coef_0$ = 116	-	-
SVM rad. k.	$\gamma = 2.7*10^{-2}$	$\gamma = 1.0*10^{-1}$	$\gamma = 1.7*10^{-2}$	-	-
SVM sigm. k.	$\gamma = 4.7*10^{3}$, $coef_0$ = 107	$\gamma = 2.3*10^{2}$, $coef_0$ = -48	$\gamma = 3.1*10^{2}$, $coef_0$ = 307	-	-
XGBoost	rounds = 84, γ = 0, $\eta = 4.1*10^{-2}$, depth = 5	rounds = 67, $\gamma = 1.0*10^{-1}$, $\eta = 3.6*10^{-1}$, depth = 4	rounds = 78, γ = 0, $\eta = 9.0*10^{-3}$, depth = 3	rounds = 329, $\gamma = 2.9*10^{-1}$, $\eta = 1.0*10^{-2}$, depth = 6	rounds = 614, γ = 1.7, $\eta = 6.5*10^{-4}$, depth = 6
MLP	h. layers = 1, nodes = 88, drop rate = 0.01	h. layers = 1, nodes = 29, drop rate = 0.21	h. layers = 2, nodes = [80,40] drop rate = 0.36	h. layers = 1, kernels = 201 drop rate = 0.28	h. layers = 1, kernels = 133, drop rate = 0.79
CNN	c. layers = 1, kernels = 299, k. size = 2	c. layers = 4, kernels = [12, 30, 32, 38], k. size = [1, 1, 1, 1]	c. layers = 4, kernels = [102, 101, 62, 291] k. size = [2, 5, 3, 2]	c. layers = 1, kernels = 244, k. size = 9,	c. layers = 1, kernels = 202, k. size = 2
ResNet	blocks = 1, b. kernels = 30, b. k. size = 1, c. kernels = 82, c. k. size = 17	blocks = 4, b. kernels = [53, 67, 52, 47], b. k. size = [1, 1, 1, 1], c. kernels = 39, c. k. size = 4	blocks = 4, b. kernels = [10, 20, 30, 40], b. k. size = [2, 2, 3, 3], c. kernels = 30, c. k. size = 3	blocks = 5, b. kernels = [509, 378, 141, 79, 9], b. k. size = [4, 4, 3, 3, 2] c. kernels = 771, c. k. size = 2	block = 2, b. kernels = [80, 89], b. k. size = [1, 4], c. kernels = 52, c. k. size = 1
	Mann-Whitney				PLS-DA
SVM pol	degree = 4, $\gamma = 2.4*10^{-7}$, $coef_0$ = -12	degree =1.8, $\gamma = 1.0*10^{6}$, $coef_0$ = 16	degree = 3, $\gamma = 1.0*10^{3}$, $coef_0$ = 100	-	-
SVM rad	$\gamma = 5.1*10^{-4}$	γ = 1.6	$\gamma = 4.2*10^{-2}$	-	-
SVM sigm	$\gamma = 3.1*10^{5}$, $coef_0$ =62	$\gamma = 1.5*10^{2}$, $coef_0$ = 147	$\gamma = 1.0*10^{3}$, $coef_0$ = 100	-	-
Xgboost	rounds = 103, $\gamma = 1.1*10^{-2}$, $\eta = 7.1*10^{-3}$, depth = 2	rounds = 205, γ = 0, $\eta = 1.9*10^{-1}$, depth = 2	rounds = 102, γ = 1.34, $\eta = 1.1*10^{-2}$, depth = 5	rounds = 407, γ = 0, $\eta = 9.1*10^{-3}$, depth = 3	rounds = 241, γ = 0, $\eta = 5.7*10^{-3}$, depth = 2
MLP	h. layers = 3, nodes = [13, 33, 38], drop rate = 0.96	h. layers = 1, nodes = 90, drop rate = 0.34	h. layers = 1, nodes = 39, drop rate= 0.25	h. layers = 1, nodes = 186, drop rate = 0	h. layer = 1, nodes = 106, drop rate = 0.26
CNN	c. layers = 1, kernels = 148, k. size = 2	c. layers = 1, kernels = 299, k. size = 2	c. layers =4, kernels = [180, 150,120, 90], k. size = [3,3,3,3]	c. layers = 1, kernels = 100, k. size = 3	c. layer = 1, kernels = 31, k. size = 2

ResNet	blocks = 1,<br>b. kernels = 496,<br>b. k. size = 3,<br>c. kernels = 402,<br>c. k. size = 6	blocks = 6,<br>b. kernels =[236, 127, 307, 164, 21, 326],<br>b. k. size = [4, 3, 4, 1, 1,2],<br>c. kernels =354,<br>c. k. size= 1	blocks = 4,<br>b. kernels = [323, 88, 122, 91],<br>b. k. size = [4, 3, 4, 2],<br>c. kernels = 314,<br>c. k. size = 1	blocks = 11,<br>b. kernels = [66, 29, 42, 9, 59, 49, 55, 50, 52, 63, 29],<br>b. k. size = [1, 2, 1, 2, 3, 2, 2, 2, 2, 2, 2],<br>c. kernels = 13,<br>c. k. size = 1	blocks = 1,<br>b. kernels = 398,<br>b. k. size = 3,<br>c. kernels = 329,<br>c. k. size = 2

Table S5. Model accuracy based on binary marker sets and final one-vs-one (OvO) classification. The highest accuracy values are highlighted in bold.

Method	Benign vs malignant	Benign vs control	Malignant vs control	One-versus-One multiclass
SVM-REF				
Naive Bayes	0.60	0.73	0.86	0.61
OPLS-DA	0.63	0.94	0.9	0.67
Random Forest	0.65	0.92	**0.92**	0.68
SVM, lin. k	0.68	0.92	0.88	0.67
SVM, pol. k	0.65	0.92	0.9	0.66
SVM, rad. k	0.68	0.92	**0.92**	0.72
SVM, sigm. k	0.60	0.75	0.64	0.55
XGBoost	0.71	**0.96**	0.9	0.74
MLP	0.69	0.81	0.82	0.61
CNN	0.71	0.92	0.86	0.71
ResNet	0.74	0.88	0.8	0.68
Mann-Whitney				
Naive Bayes	0.69	0.85	0.88	0.68
OPLS-DA	0.60	0.85	0.82	0.60
Random Forest	0.63	0.90	0.90	0.66
SVM, lin. k	0.56	0.85	0.82	0.59
SVM, pol. k	0.35	0.85	0.90	0.46
SVM, rad. k	0.66	0.92	0.90	0.70
SVM, sigm. k	0.47	0.75	0.80	0.50
XGBoost	0.69	0.88	**0.92**	0.73
MLP	0.71	0.83	0.84	0.67
CNN	**0.81**	0.85	0.88	**0.77**
ResNet	0.76	0.92	0.88	0.76

Table S6. Mean recall of models based on binary marker sets and accuracy of final one-vs-one (OvO) classification. The best results are highlighted in bold.

Method	Benign vs Malignant	Benign vs Control	Malignant vs Control	One-versus-One multiclass
SVM-REF				
Naive Bayes	0.59	0.75	0.87	0.64
OPLS-DA	0.63	0.93	0.91	0.69

Random Forest	0.64	0.90	**0.92**	0.69
SVM, lin. k	0.68	0.93	0.89	0.70
SVM, pol. K	0.64	0.93	0.91	0.68
SVM, rad k	0.68	0.91	0.93	0.73
SVM, sigm. K	0.60	0.73	0.64	0.55
XGBoost	0.71	**0.95**	0.91	0.76
MLP	0.69	0.83	0.83	0.65
CNN	0.71	0.94	0.88	0.74
ResNet	0.74	0.89	0.83	0.71
Mann-Whitney				
Naive Bayes	0.69	0.86	0.88	0.70
OPLS-DA	0.59	0.83	0.83	0.61
Random Forest	0.62	0.88	0.88	0.67
SVM, lin. k	0.56	0.85	0.82	0.61
SVM, pol. k	0.36	0.85	0.89	0.51
SVM, rad. K	0.66	0.90	0.89	0.70
SVM, sigm. K	0.47	0.73	0.80	0.52
XGBoost	0.69	0.88	**0.92**	0.75
MLP	0.70	0.84	0.84	0.69
CNN	**0.81**	0.88	0.88	**0.78**
ResNet	0.76	0.93	0.88	**0.78**

Table S7. Performance evaluation (accuracy and mean recall) of machine learning models comparing feature selection strategies for binary and multiclass classification. Best results are highlighted in bold.

Method	Accuracy				Recall			
	SVM-REF	Kruskall-Wallis	Mann-Whitney	PLS-DA	SVM-REF	Kruskall-Wallis	Mann-Whitney	PLS-DA
Naive Bayes	0.66	0.72	0.67	0.60	0.67	0.73	0.68	0.62
PLS-DA	0.56	0.62	0.61	0.59	0.60	0.63	0.62	0.63
Random Forest	0.72	0.68	0.67	0.67	0.73	0.69	0.68	0.68
XGBoost	0.72	**0.77**	0.74	0.68	0.73	**0.78**	0.75	0.70
MLP	0.72	0.76	0.76	0.66	0.73	**0.78**	0.77	0.69
CNN	0.73	0.74	0.73	0.66	0.74	0.77	0.75	0.69
ResNet	0.71	0.72	0.72	0.68	0.72	0.74	0.74	0.33

Table S8. Features with statistical significant association with patients age, coefficient of association and significance of association (Spearman's test).

Features	R	p
Alanine	-0.16	0.02
Arginine	-0.19	0.004
CE 18:3	-0.14	0.03
Cer-NDS d18:0/24:0	-0.16	0.02
CerP d18:0/26:0	-0.19	0.003
Citrate	-0.17	0.01

DG 16:0_18:1	0.2	0.002
DG 18:1_18:1	0.22	0.001
Glutamine	-0.14	0.04
Glycine	-0.17	0.01
HexCer-NS d18:1/22:0	-0.2	0.003
LPC 16:0	-0.16	0.01
LPC 16:1	-0.15	0.03
LPC 18:0	-0.19	0.004
LPC 18:1	-0.16	0.02
LPC 18:2	-0.25	<0.001
LPC 20:4	-0.18	0.006
LPC 20:5	-0.19	0.003
LPC 22:6	-0.14	0.03
LPE 18:0	-0.19	0.004
LPE 18:2	-0.18	0.008
Lysine	-0.15	0.03
MGDG 18:1_22:6	0.22	0.001
n-2-Aminobutyrate	-0.17	0.01
OxCL 16:0_18:2_20:4(OOH)2_22:6	0.16	0.01
OxLPC 18:3(OOO)	-0.15	0.02
OxPC 16:0_22:3(OH)	0.15	0.03
OxPC 16:1_18:2(OOO)	-0.15	0.02
OxPC 18:0_18:4(Ke,OH)	0.2	0.003
OxPC 18:1_16:1(COOH)	-0.17	0.01
OxPC 18:2_16:1(COOH)	-0.15	0.02
OxPE 16:0_18:2(OOO)	0.23	0.001
OxPG 18:1_20:3(1O)	-0.19	0.005
OxTG 16:0_18:1_16:1(CHO)	0.15	0.02
OxTG 16:1_18:0_16:1(COOH)	-0.17	0.009
PC 14:0_18:2	-0.14	0.03
PC 14:0_20:4	-0.13	0.046
PC 16:0_16:0	0.21	0.001
PC 16:0_18:1	0.14	0.04
PC 16:0_18:3	0.29	<0.001
PC 16:0_20:2	-0.15	0.02
PC 16:1_18:4	-0.14	0.03
PC 18:2_20:3	-0.16	0.02
PC 18:2_22:6	-0.2	0.003
PC 20:4_22:6	-0.13	0.048
PE 16:0_22:6	0.28	<0.001
PE 18:1_20:0	0.23	0.001
PG 18:0_20:5	-0.15	0.02
LPC O-16:0	-0.23	<0.001
LPC O-16:1	-0.2	0.003
LPC O-18:1	-0.15	0.03
PC O-16:0/20:4	-0.18	0.005
PC O-16:1/18:1	-0.23	<0.001

PC O-16:1/18:2	-0.16	0.02
PC O-18:0/18:2	-0.16	0.01
PC O-18:0/20:4	-0.16	0.01
PC O-20:0/18:1	-0.15	0.02
PC O-22:0/18:2	0.19	0.003
LPC P-16:0	-0.23	0.000
PC P-16:0/18:2	-0.16	0.01
PC P-18:0/18:1	-0.21	0.001
PC P-18:0/18:2	-0.17	0.01
PC P-18:1/20:4	-0.16	0.02
PC P-20:0/18:1	-0.18	0.006
PC P-20:0/18:2	-0.2	0.003
PC P-22:1/18:2	-0.15	0.02
PE P-16:0/20:4	-0.16	0.01
PE P-18:0/18:2	-0.21	0.001
PS P-20:0/18:1	-0.17	0.01
PS 16:0_20:3	0.28	<0.001
Serine	-0.14	0.03
SM d14:0/26:0	-0.18	0.01
SM d16:0/18:0	0.19	0.005
SM d16:1/14:0	-0.15	0.03
SM d18:1/18:0	0.23	<0.001
SM d18:1/20:0	-0.14	0.04
SM d18:1/22:0	-0.16	0.01
SM d18:1/22:1	-0.15	0.02
SM d18:2/24:0	0.16	0.02
SM d20:0/16:0	0.23	<0.001
SM d20:0/16:1	0.2	0.003
SM d20:0/18:1	-0.13	0.049
SM d22:0/18:1	-0.15	0.02
SM d22:0/18:2	-0.17	0.01
TG 10:0_16:1_22:6	-0.19	0.004
TG 10:0_18:2_18:2	-0.17	0.01
TG 10:0_18:2_22:6	0.17	0.01
TG 12:0_18:2_18:3	0.16	0.02
TG 16:0_16:0_18:1	0.17	0.01
TG 16:0_18:0_18:1	0.2	0.002
TG 16:0_18:0_18:4	-0.16	0.01
TG 16:0_18:1_18:1	0.21	0.001
TG 16:0_18:1_22:5	0.17	0.01
TG 16:0_18:1_22:6	0.17	0.01
TG 16:1_18:0_18:1	0.19	0.004
TG 16:1_22:4_8:0	-0.14	0.03
TG 18:0_18:1_18:1	0.19	0.003
TG 18:0_18:1_18:2	0.23	<0.001
TG 18:0_18:2_20:3	0.16	0.02
TG 18:0_22:6_8:0	0.16	0.02

TG 18:1_18:1_20:5	0.14	0.03
TG 18:1_18:1_22:6	0.14	0.03
TG 18:1_18:2_22:6	0.16	0.02
Tyrosine	-0.16	0.02
Valine	-0.14	0.03
